# A Rare Case of Dose-Dependent Losartan-Induced Angioedema

**DOI:** 10.7759/cureus.24110

**Published:** 2022-04-13

**Authors:** Bilal A Niazi, Manpreet Kaur, Maurice Mosseri, Abraham Lo

**Affiliations:** 1 Internal Medicine, Hackensack Meridian Health - Palisades Medical Center, North Bergen, USA

**Keywords:** arb, bradykinin, dose-dependent, losartan, angioedema

## Abstract

Angiotensin converting enzyme inhibitors (ACE-Is) have long been associated with angioedema and cough. These complications are thought to be related to an increase in bradykinin levels. Angiotensin receptor blockers (ARBs) such as losartan, however, are not known to increase bradykinin levels and, therefore, this complication is not as widely recognized. However, there is a significant proportion of patients who develop angioedema on ARB medications after previous episodes of angioedema on ACE-I. Though there is increasing literature to support that the patients may develop angioedema while taking ARBs such as losartan, a dose-dependent nature has not been well documented. We present a patient with a 20-year history of losartan use who developed angioedema suddenly after an increase in dosage. A dose-dependent relationship between ARBs and angioedema has not been well documented and this is the first documented case of angioedema presenting in a dose-dependent manner with losartan use. We hope that our case will bring awareness to the potential dose-dependent relationship between losartan and angioedema in order to aid clinicians when titrating ARB medications in order to expediently diagnose the fatal side-effect of angioedema and to encourage further research.

## Introduction

Angiotensin receptor blockers (ARBs) are medications that block angiotensin II from binding to the angiotensin II receptor in vascular smooth muscles and adrenal gland tissues. In comparison to angiotensin converting enzyme inhibitors (ACE-Is), ARBs do not interfere with the synthesis of angiotensin II. Additionally, ACE-Is, unlike ARBs, are known to inhibit the degradation of bradykinin leading to vasodilation, increased vascular permeability, and angioedema [1]. In the past, meta-analyses have shown that the risk of angioedema from an ARB is minimally increased when compared to a placebo [2]. In fact, once patients present with ACE-I induced angioedema, patients should be switched to an ARB four to six weeks after discontinuation of ACE-I, with cross reactivity noted to be less than 10% [3]. In this case report, we present a case of ARB-induced angioedema after an increase in losartan dosage. A dose-dependent relation with ARBs, especially losartan, has not been well documented. We hope to bring awareness of this potential dose-dependent relationship to clinicians when titrating ARB medications in order to expediently diagnose the potentially fatal outcomes from angioedema. 

## Case presentation

Here we present an 87-year-old patient with a history of asthma and congestive heart failure who presented for shortness of breath, which started suddenly three to four hours prior to arrival to the emergency department. He had been resting at home prior to the onset of his symptoms and could not recall any possible precipitating factors. He tried using his albuterol inhaler without any relief of his symptoms. His home medications included losartan, hydralazine, and torsemide. He had reported taking losartan 100 mg for over 20 years. He denied any previous history of ACE-I use. On arrival to the emergency room, his vitals were unremarkable and his physical exam was remarkable for expiratory wheezing, without any significant respiratory distress, lower extremity edema, elevated jugular vein distention (JVD), urticaria, pruritus, lip swelling, or changes in voice. Laboratory evaluation was unremarkable and chest X-ray conducted did not reveal any infiltrates, edema, or effusions. Ultimately he was administered magnesium and started on bronchodilators for management of possible asthma exacerbation. No steroids were administered or started. His shortness of breath and expiratory wheezing progressively improved and his losartan was increased on day 3 of admission from 100 to 200 mg for poorly controlled hypertension.

On day 4 of his admission, his shortness of breath suddenly worsened. On evaluation, he was found to have periorbital swelling without associated erythema, urticaria, or pruritus. He was not in any respiratory distress and was not found to have changes to his voice or wheezing. No new medications or interventions were started prior to the onset of his symptoms except for an increase in losartan dose. No supplementary oxygen was required as he was saturating greater than 97% on room air. He was administered diphenhydramine with minimal relief and losartan was discontinued. Hydralazine was increased and nifedipine was added for blood pressure management. Further laboratory evaluation did not reveal any significant changes. On further evaluation, his shortness of breath improved by the next day with markedly decreased periorbital swelling. By the second day after the onset of his periorbital edema, his swelling had completely resolved.

## Discussion

Angioedema is a self-limited process that causes swelling of the lips, tongue, eyes, and even larynx [4]. Our patient presented with periorbital swelling without urticaria or pruritus after a dosage increase of losartan. Histamine and bradykinin are the most commonly implicated peptides in angioedema and work by increasing vascular permeability. The bradykinin variant of angioedema can be differentiated from the histamine variant in that it is less likely to cause urticaria or pruritis and it is more likely to be resistant to steroids and anti-histaminergic agents [5].

**Figure 1:**
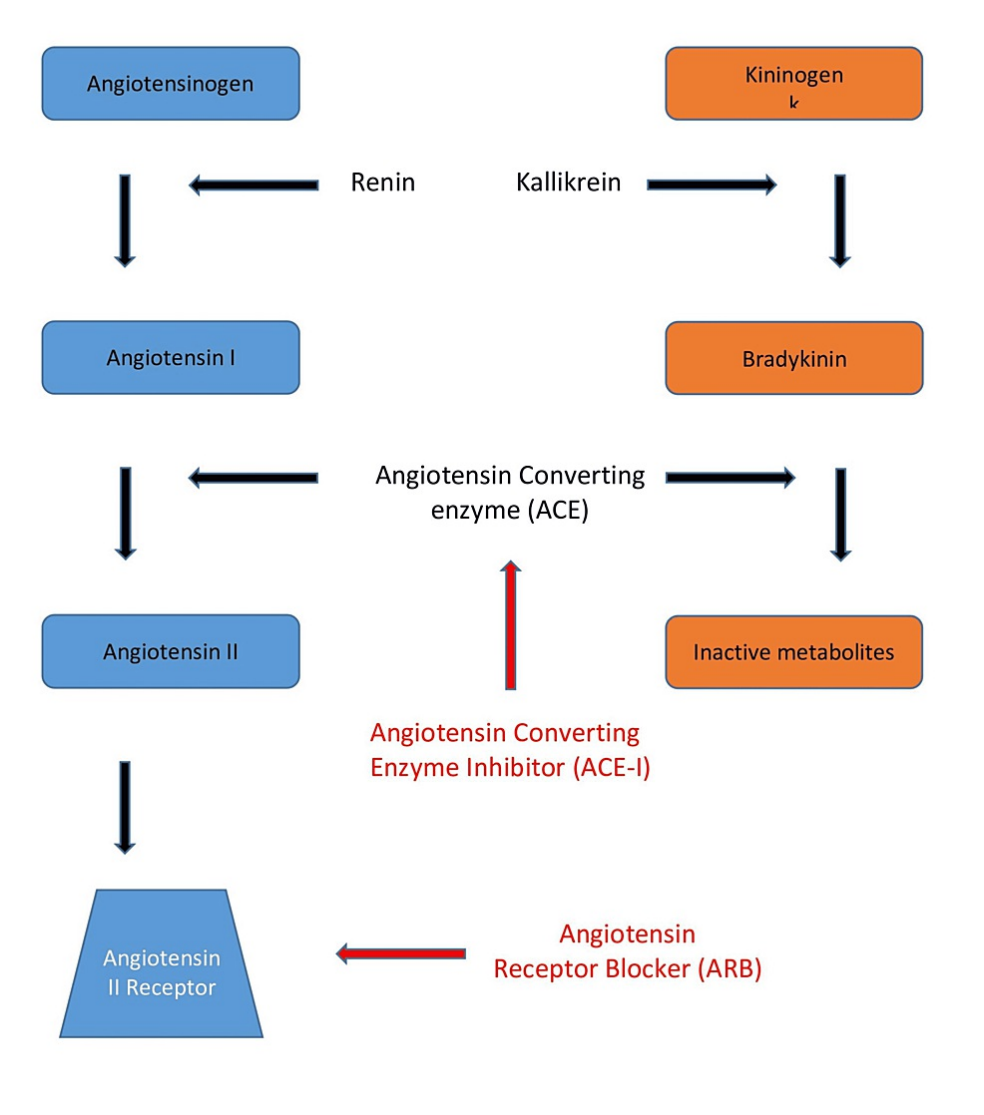
Angiotensin and bradykinin metabolic pathway. This figure displays how ACE-I and ARBs function on the metabolic pathway of angiotensin as well as highlights the action of ACE on the bradykinin breakdown pathway. Enzymes and peptides in red reflect an inhibitory effect. Image credit: Bilal Niazi. ACE-I, angiotensin converting enzyme inhibitors; ARBs, angiotensin receptor blockers

Bradykinin-induced angioedema can be seen in hereditary C1 esterase inhibition, acquired C1 esterase deficiency, and with ACE-I medications [6]. The ACE-I medication class appears to be the most common cause of angioedema. In fact, ACE-Is have been implicated in up to one-third of emergency visits for angioedema [7]. ACE-I work by decreasing the conversion of angiotensin I to angiotensin II, resulting in decreased systemic vasoconstriction. For these purposes, ACE-I medications are commonly used to manage hypertension. However, ACE also functions to break down the peptide bradykinin, which prevents excessive vascular permeability [8]. Subsequently, ACE-I can result in elevated levels of the peptide bradykinin through the inhibition of ACE, as shown in Figure 1, potentially leading to increased vascular permeability and angioedema.

The ARBs are an alternative to ACE-I medications which can be used for the management of hypertension when an ACE-I is not tolerated. Like ACE-Is, ARBs work on the renin-angiotensin-aldosterone pathway to blunt the effect of angiotensin II. ARBs function as a receptor blocker to angiotensin II, whereas ACE-I inhibits the conversion of angiotensin I to angiotensin II. Additionally, ARBs do not affect the bradykinin pathway, so it would be unlikely to see elevated levels of bradykinin with the use of ARBs as opposed to ACE-I use. In fact, angioedema related to ARBs is quite rare and its mechanism of action is not well understood. However, it has been reported that up to 32% of patients with ARB-induced angioedema had previously experienced ACE-I-related angioedema [9]. This relationship may suggest that bradykinin plays a role in ARB-induced angioedema through an unknown mechanism.

Angiotensin converting enzyme inhibitors appear to have a dose-dependent relationship with the incidence of angioedema [10]. This is thought to be due to a subsequent dose-dependent elevation in bradykinin levels. Though, it is poorly understood how a dose-dependent relationship of an ARB could affect the incidence of angioedema. Although our patient had not previously used an ACE-I, cross-reactivity of ARBs and ACE-I on angioedema could suggest that ARBs have a dose-dependent relationship with angioedema like ACE-Is by variably increasing bradykinin levels.

## Conclusions

Though ARB-induced angioedema has been infrequently documented, a dose-dependent relationship of ARBs on angioedema has been even less documented. In particular, our finding is the first documented case of a dose-dependent relationship of losartan and angioedema. Due to the cross-reactivity of ACE-I and ARBs as well as the dose-dependent relationship between ACE-I and angioedema, it is likely that elevated bradykinin levels contribute to ARB-related angioedema in a dose-dependent manner. Angioedema is a potentially fatal outcome, which requires early and immediate identification. Additionally, ARBs are commonly prescribed medications that are frequently titrated. We hope to bring awareness to the potentially fatal dose-dependent relationship of ARBs and angioedema to clinicians when titrating ARB medications as well as to add to the growing body of literature and encourage further research.
